# Metal Ion Supplementation to Boost Melanin Production by *Streptomyces nashvillensis*

**DOI:** 10.3390/ijms26010416

**Published:** 2025-01-06

**Authors:** Odile Francesca Restaino, Talayeh Kordjazi, Francesco Tancredi, Paola Manini, Fabiana Lanzillo, Francesca Raganati, Antonio Marzocchella, Raffaele Porta, Loredana Mariniello

**Affiliations:** 1 Department of Chemical Sciences, Università degli Studi di Napoli Federico II, Monte sant’Angelo Campus, Via Cintia 4, 80126 Naples, Italy; talayeh.kordjazi@unina.it (T.K.); francesco.tancredi01@gmail.com (F.T.); pmanini@unina.it (P.M.); raffaele.porta@unina.it (R.P.); loredana.mariniello@unina.it (L.M.); 2 Department of Chemical, Materials and Production Engineering, Università degli Studi di Napoli Federico II, P. le V. Tecchio 80, 80125 Naples, Italy; fabiana.lanzillo@unina.it (F.L.); francesca.raganati@unina.it (F.R.); marzocch@unina.it (A.M.)

**Keywords:** copper, eumelanin, iron, L-tyrosinase, *Streptomyces nashvillensis*

## Abstract

As Streptomycetes might produce melanin to survive in stressful environmental conditions, like under metal exposure, supplementing metal ions to the growth medium could be a wise strategy for boosting the production of the pigment. The aim of this study was to test, for the first time, the possibility of boosting *S. nashvillensis* DSM40314 melanin biosynthesis by adding to the growth medium singularly or, at the same time, different concentrations (1.0, 1.5, and 2.0 g∙L^−1^) of CuSO_4_ or/and Fe_2_(SO_4_)_3_. A maximum melanin production of 4.0 ± 0.1 g·L^−1^ was obtained in shake flasks with a 2.0 g∙L^−1^ coupled addition of the two metals, while the extracellular tyrosinase activities ranged values between 5.4 and 11.6 ± 0.1 U·L^−1^. The pigments produced in different conditions were precipitated from the broth supernatants under acidic conditions, purified, and characterized by UV-VIS, FT-IR, and NMR analyses that determined structures like eumelanin pigments. Fermentation experiments in stirred tank reactors allowed to scale up the process in more controlled conditions, further boosting the pigment production up to 4.9 ± 0.1 g·L^−1^, with an increase of about 22.0% compared to the results obtained in shake flasks.

## 1. Introduction

Melanins are pigments found in different domains. Their colors vary from reddish to dark brown, and they show distinctive physical-chemical characteristics, like good UV-visible light absorption, high thermal stability, anti-microbial and antioxidant activities, redox properties and capacity of chelating metal ions, and hybrid ionic-electronic conductance, as well as biocompatibility, low cytotoxicity and no antigenic response [1,2]. Thanks to their properties, they are conventionally used as pigments in food industries, cosmetics, and textile products, while, more recently, they have also been employed in biomedical applications, bioremediation procedures, and bioplastic preparation [3,4]. Nowadays, melanin is manufactured through extraction from the ink sacs of cephalopods, like cuttlefish (*Sepia officinalis*), with expensive, environmentally unfriendly, and not sustainable processes that are mainly dependent on the animal supply availability [5], or alternatively, it is obtained through procedures of chemical synthesis [6]. The biotechnological production of melanin by microorganisms might represent a sound alternative that is sustainable and easy to scale up. However, to be economically reliable, the biotechnological process must ensure high production values (at least melanin concentrations in the range of g·L^−1^) and high productivity by employing cheap strategies to boost the yield and diminish the timing of the process at the same time. To move from laboratory research to an effective industrialization of the process, physiological studies are first needed after the identification of suitable strains, a robust and reproducible fermentation process should be set upby using low-cost nutrients, and then scale-up studies are necessary. Moreover, as the purification step is the costliest part of a biotechnological process, simple downstream procedures should be designed [4,7,8]. Between the microorganisms, fungi like *Auricularia*, *Aspergillus*, *Armillaria*, *Cryptococcus*, and *Pletorus* and bacteria species like *Proteus*, *Pseudomonas*, and *Streptomyces* have been identified as being able to produce different types of melanin as pigments to protect themselves from environmental harsh conditions like high temperatures, UV, and visible light or metal exposure [9]. However, compared with fungi, *Streptomyces* produce melanin in a shorter time (about 120–168 h versus about 336 h), and as an extracellular pigment, so that it is then easier to recover and purify [4]. However, so far, very few studies have explored innovative biotechnological strategies for increasing the *Streptomyces* pigment production ability. In the strains that have a DOPA pathway, the melanin synthesis starts thanks to the activity of a tyrosinase enzyme (E.C. 1.14.18.1), an extracellular released oxidoreductase with a binuclear copper (II) center that catalyzes both the ortho hydroxylation of the monophenols to the o-diphenols (monophenolase activity) in the presence of oxygen and the following oxidation of the o-diphenols to the o-quinones (di-phenolase activity) [10]. Thus, tyrosinase first converts L-tyrosine into L-3,4-dihydroxyphenylalanine (L-DOPA) and then L-DOPA into DOPA-quinone, the precursor of dopachrome, which is then oxidated into 5,6-dihydroxyindole (DHI) and 5,6 dihydroxyindole-2-carboxylic acid (DHICA). These units are then randomly polymerized to obtain a brown or black eumelanin. Dopachrome might, in alternative, also be converted into 5-S-cysteinyldopa, the precursor of a yellow or red pheomelanin [3,9,11,12]. The activation of tyrosinase from its apotyrosinase form and its secretion depends on the copper-ion ligand presence [13,14,15,16,17,18]. So far, from a biotechnological process point of view, literature data have only demonstrated that melanin production depends on the bacterial physiological state, the growth conditions (e.g., pH and temperature), and the nutrients availability, similarly to other metabolites synthesized by *Streptomyces* strains [19]. The reported pigment concentrations varied in shake flasks from 0.09 g∙L^−1^ to 3.94 g∙L^−1^, according to the strain, the temperature (in the range 26–40 °C) and the pH of growth (in the range 6.0–8.0), and the different medium components [4]. Complex nutrients like dextrose, starch, malt extract, yeast extract, and soy peptone, as well as simple amino acids like L-tyrosine, have been explored as carbon and nitrogen sources to formulate growth media suitable to boost melanin production [4,7,20,21,22]. More recently, lignocellulose wastes, like *Posidonia oceania* egagropili or fruit extracts, have been tested as substrates for pigment production by *Streptomyces* in the perspective of more sustainable biotechnological production processes [23,24]. Instead, the supplementation of metal ions to boost melanin synthesis by providing co-factors useful for the biosynthetic machinery has been scarcely investigated. In general, copper (II) or iron (both II and III) salts have been employed in low concentrations, between 0.00000027 and 0.5 g∙L^−1^, to formulate growth media of different *Streptomyces* strains [25,26,27,28,29,30,31,32], without specifically testing their effect on the pigment production. In only one study, the effect of one single concentration of iron (II) salt (as 1.0 g∙L^−1^ FeSO_4_) or of nickel (II) salt (0.25 g∙L^−1^ NiCl_2_) was specifically explored for melanin production by *Streptomyces* sp. ZL-24 [33]. No study has investigated the eventual addition of different concentrations of copper (II) salts to the growth medium to help the catalytic activity of the tyrosinase enzyme, nor its coupled supplementation with other metals. The presence of copper ions in the growth medium would help melanin production for several reasons. As tyrosinase is an enzyme with a binuclear copper (II) center, the supplementation of this metal ion in the growth medium might help to increase its action and to more easily convert L-tyrosine into L-3,4-dihydroxyphenylalanine (L-DOPA) and then L-DOPA into DOPA-quinone, the precursors of the melanin pigment [10]. Moreover, as tyrosinase is initially expressed in its apoform, the presence of copper ions in the medium would help the transformation to its active conformation [10].

A previous paper, representing the first physiological study, proved that the strain *Streptomyces nashvillensis* DSM40314 might produce a eumelanin-like pigment in shake flasks, up to 0.74 ± 0.01 g·L^−1^, in 96 h when grown on a medium containing glucose, yeast extract, and malt extract at 28 °C, pH 7.0, and 250 rpm [34]. The aim of this study was to explore, for the first time, the possibility of boosting melanin production by *Streptomyces nashvillensis* by adding to the growth medium singularly or, at the same time, different concentrations (1.0, 1.5, and 2.0 g∙L^−1^) of CuSO_4_ or/and Fe_2_(SO_4_)_3_. Experiments were first performed in shake flasks, and the cellular growth, melanin production, and extracellular tyrosinase activity were evaluated. The pigments, produced extracellularly in the different conditions, were then purified and characterized by UV-visible absorbance, FT-IR, and mono- and bidimensional NMR analyses to determine their structures. To enhance the process under more controlled conditions and potentially maximize pigment synthesis, fermentation experiments were conducted utilizing controlled stirred tank reactors (STRs). These trials included the addition of 2.0 g∙L^−1^ CuSO_4_ plus 2.0 g∙L^−1^ Fe_2_(SO_4_)_3_ to the medium as supplementary components. This experimental setup aimed to facilitate the scale-up of the process while providing a more regulated environment for investigation.

## 2. Results

### 2.1. Shake Flask Experiments

#### 2.1.1. Shake Flask Experiments with Single Supplementations of Fe_2_(SO_4_)_3_ or CuSO_4_

**Figure 1:**
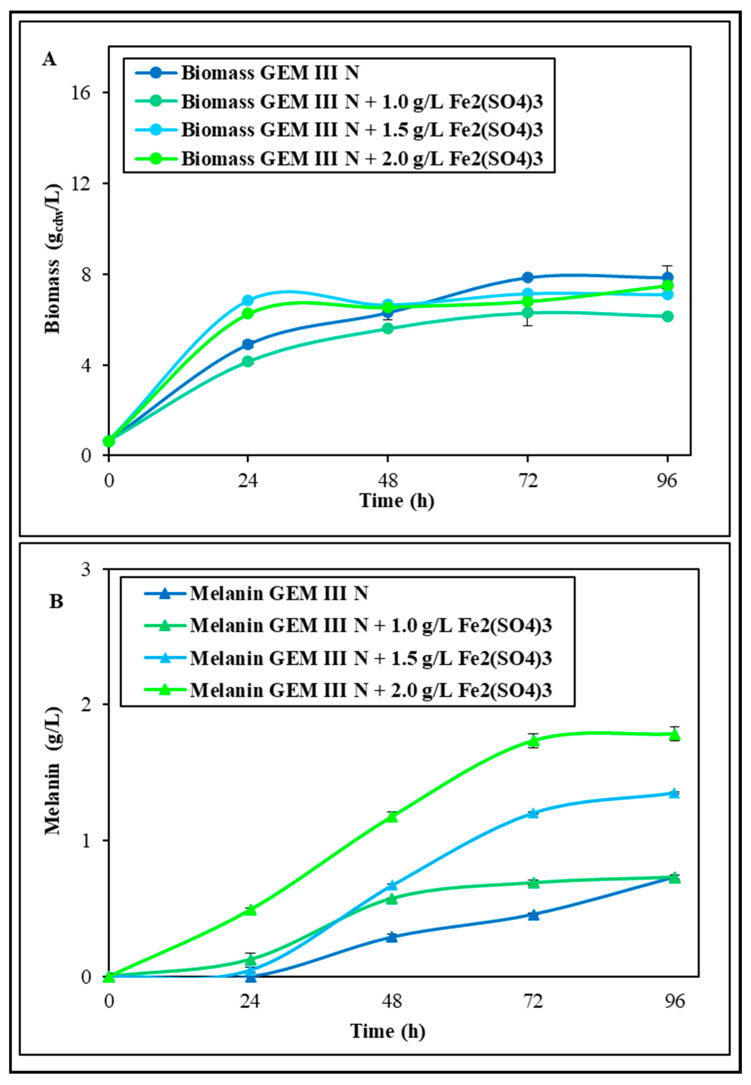
*S. nashvillensis* growth (**A**) and melanin production (**B**) in 96 h shake flask runs on GEM III N medium added with different concentrations of Fe_2_(SO_4_)_3_ (from 1.0 to 2.0 g∙L^−1^) compared with the control.

**Figure 2:**
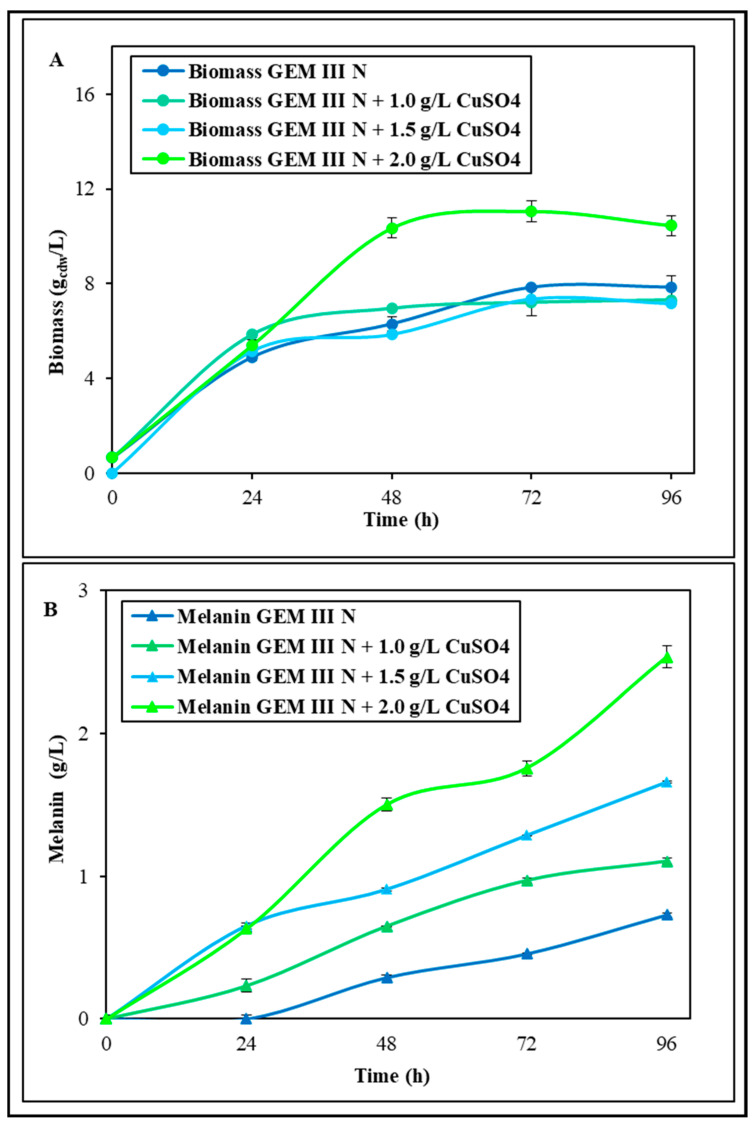
*S. nashvillensis* growth (**A**) and melanin production (**B**) in 96 h shake flask runs on GEM III N medium added with different concentrations of CuSO_4_ (from 1.0 to 2.0 g∙L^−1^) compared with the control.

The first shake flask experiments were performed to test the effect of the medium supplementation with Fe_2_(SO_4_)_3_ or CuSO_4_ on both growth and melanin production (Figure 1 and Figure 2). Different concentrations (1.0, 1.5, and 2.0 g∙L^−1^) of each salt were added, and all the experiments were performed at 28 °C and pH 7.0 in comparison with a control whose final biomass and melanin values were 7.9 ± 0.2 g_cdw_·L^−1^ and 0.74 ± 0.01 g·L^−1^ in 96 h runs, respectively. These values of the control were similar to data previously reported [24]. In the experiments supplemented with Fe_2_(SO_4_)_3,_ when 1.0 g∙L^−1^ was added, the bacterial growth resulted similar to that of the control growth, while with 1.5 and 2.0 g∙L^−1^ supplementation, the growth resulted increased from 28.7 to 33.6% in the first 24 h (Figure 1A). However, similar final biomass values were noted in all three conditions, ranging from 6.2 ± 0.1 to 7.5 ± 0.1 g_cdw_·L^−1^, thus even 22.0% lower than the control. Melanin production, instead, was greatly influenced by the Fe_2_(SO_4_)_3_ supplementations with boosted values at 96 h in all three conditions: the final concentrations were 0.89 ± 0.01, 1.10 ± 0.01, and 1.71 ± 0.01 g∙L^−1^, which were thus about 1.2-, 1.5-, and 2.3-fold higher than the control, respectively (Figure 1B). With the 2.0 g∙L^−1^ Fe_2_(SO_4_)_3_ supplementation, the maximum yield on biomass (0.24 ± 0.01 g·g_cdw_^−1^) and productivity were also obtained (0.018 ± 0.001 g·L^−1^·h^−1^), representing 2.7- and 2.2-fold increases compared with the values obtained in the control experiments (0.09 ± 0.01 g·g_cdw_^−1^ and 0.008 ± 0.001 g·L^−1^·h^−1^). In the experiments with CuSO_4_ addition, instead, the 1.0 g∙L^−1^ or 1.5 g∙L^−1^ concentrations did not change the bacterial growth, whichremained similar to that of the control, with the final biomass values being even 8.8% lower (both values were 7.2 ± 0.1 g_cdw_·L^−1^) (Figure 2A). The 2.0 g∙L^−1^ CuSO_4_ addition, instead, boosted the biomass since the 48th hour driving toward a final value of 10.4 ± 0.2 g_cdw_·L^−1^, which was thus 1.3-fold higher than the control. Melanin production was greatly influenced by all the concentrations of CuSO_4_ with boosted values at 96 h up to 1.10 ± 0.01, 1.66 ± 0.01, and 2.53 ± 0.01 g∙L^−1^, which were thus about 1.5-, 2.2-, and 3.4-fold higher than the control, respectively (Figure 2B). The 2.0 g∙L^−1^ CuSO_4_ supplementation also drove to the maximum yield on biomass (0.24 ± 0.01 g·g_cdw_^−1^) and productivity values (0.027 ± 0.001 g·L^−1^·h^−1^), values that were 2.7- and 3.4-fold higher than the values obtained in the control experiments (0.09 ± 0.01 g·g_cdw_^−1^ and 0.008 ± 0.001 g·L^−1^·h^−1^) (Figure 2B). As both 2.0 g∙L^−1^ of Fe_2_(SO_4_)_3_ and of CuSO_4_ resulted in a boost in melanin production, the possibility of an addictive effect was tested. (Shake flask experiments were also performed by adding NiCl_2_, but growths were five times lower than the control, with a maximum biomass value of 1.6 ± 0.2 g_cdw_·L^−1^, and no melanin production was noted).

#### 2.1.2. Shake Flask Experiments with Coupled Supplementation of Fe_2_(SO_4_)_3_ Plus CuSO_4_

**Figure 3:**
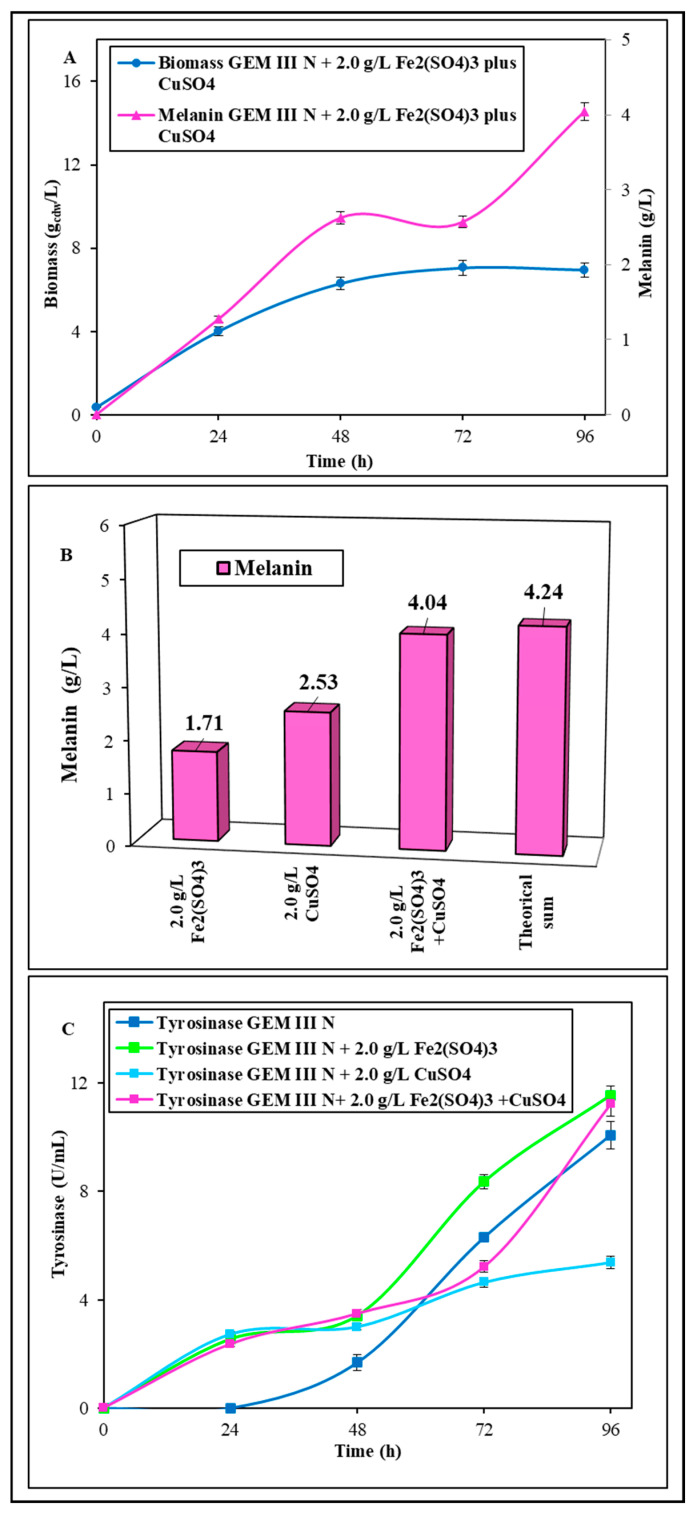
*S. nashvillensis* growth and melanin production in 96 h shake flask runs on GEM III N medium added with 2.0 g∙L^−1^ CuSO_4_ plus Fe_2_(SO_4_)_3_ (**A**). The maximum melanin values obtained experimentally with 2.0 g∙L^−1^ of Fe_2_(SO_4_)_3_ or 2.0 g∙L^−1^ CuSO_4_ or 2.0 g∙L^−1^ CuSO_4_ plus Fe_2_(SO_4_)_3_ compared with the theoretical value that might be obtained in the additive effect (**B**). Tyrosinase activities in the three best supplementation conditions compared with the control (**C**).

The possibility of an addictive effect on melanin production due to a 2.0 g∙L^−1^ Fe_2_(SO_4_)_3_ plus CuSO_4_ supplementation was further tested in shake flasks. The bacterial growth resulted like the ones observed with Fe_2_(SO_4_)_3_ supplementation, with a final biomass value of 7.0 ± 0.1 g_cdw_ g∙L^−1^ (Figure 3A), but melanin production greatly increased up to 4.04 ± 0.01 g∙L^−1^ at 96 h, which was thus 2.5- and 1.6-fold higher than the melanin concentrations obtained with the single salt addition, respectively, with a final yield on biomass of 0.58 ± 0.01 g·g_cdw_^−1^ and a productivity of 0.042 ± 0.001 g·L^−1^·h^−1^ (Figure 3A). The melanin concentration obtained with 2.0 g∙L^−1^ Fe_2_(SO_4_)_3_ plus CuSO_4_ addition resulted in a concentration only 5.7% lower than the theoretical concentration (4.23 g∙L^−1^) calculated by summing the maximum pigment production obtained with 2.0 g∙L^−1^ Fe_2_(SO_4_)_3_ or CuSO_4_ supplementation (about 1.71 and 2.53 g∙L^−1^, respectively) (Figure 3B). The activity of extracellular tyrosinase was tested in 2.0 g∙L^−1^ Fe_2_(SO_4_)_3_ plus CuSO_4_ experiments and compared to the other two best supplementation conditions (2.0 g∙L^−1^ Fe_2_(SO_4_)_3_ or CuSO_4_), as well as with the control (Figure 3C). In the first 48 h of growth, tyrosinase activity in all the supplemented experiments was always higher than the control, reaching up to 1.6-fold. With 2.0 g∙L^−1^ of Fe_2_(SO_4_)_3_ supplementation or 2.0 g∙L^−1^ Fe_2_(SO_4_)_3_ plus CuSO_4_ addition, similar final maximum tyrosinase activity values were reached up to 11.6 ± 0.1 U·L^−1^. These values were also similar to the final one of the control. In the case of CuSO_4_ supplementation, however, tyrosinase activity between 48 and 96 h was lower than that of the control, reaching a final value of 5.4 ± 0.1 U·L^−1^ (Figure 3C).

### 2.2. Melanin Purification and Characterization

**Figure 4:**
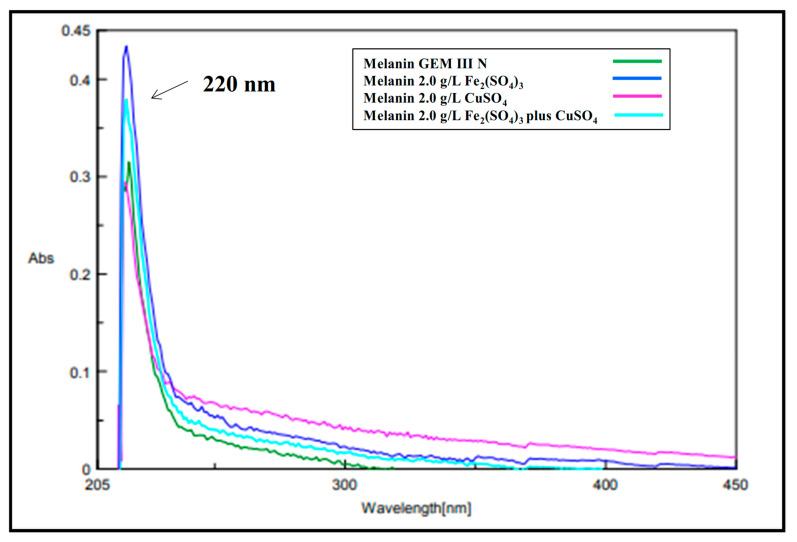
Overlaid UV absorbance spectra of the purified melanin by *S. nashvillensis* grown on GEM III N medium (green line) or on the 2.0 g∙L^−1^ Fe_2_(SO_4_)_3_- (blue line), CuSO_4_- (pink line) or CuSO_4_ plus Fe_2_(SO_4_)_3_- (light blue line) supplemented media. The maximum peak of absorbance is indicated by the arrow.

**Figure 5:**
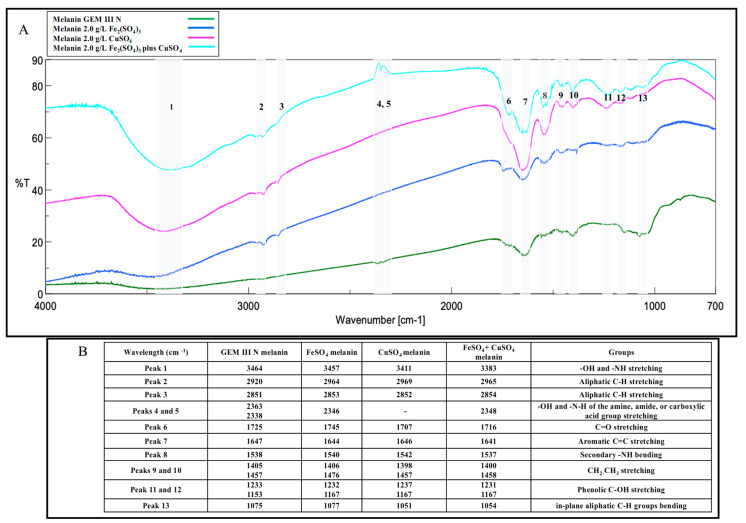
Overlaid FT-IR spectra of the purified melanin by *S. nashvillensis* grown on GEM III N medium (green line) or on the 2.0 g∙L^−1^ Fe_2_(SO_4_)_3_- (blue line), CuSO_4_- (pink line), or CuSO_4_ plus Fe_2_(SO_4_)_3_- (light blue line) supplemented media (**A**), and the wavenumbers of the different identified signals (**B**).

**Figure 6:**
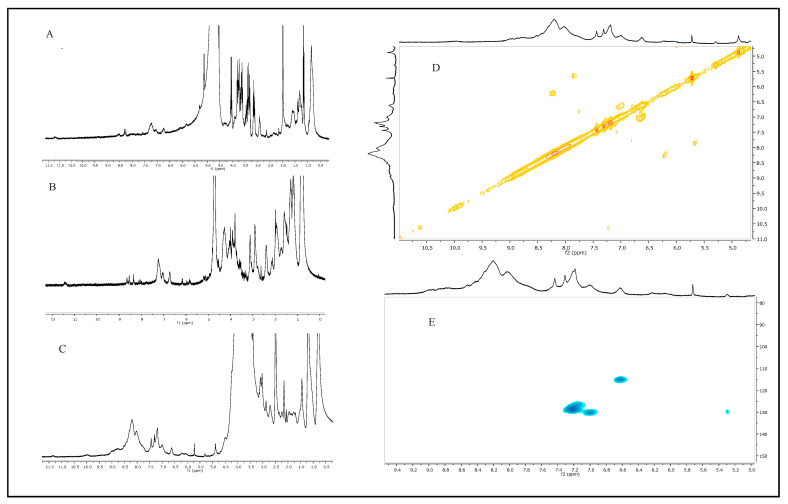
^1^H NMR spectra (DMSO-d_6_) of the purified melanin by *S. nashvillensis* grown on the 2.0 g∙L^−1^ Fe_2_(SO_4_)_3_- (**A**), CuSO_4_- (**B**), or CuSO_4_ plus Fe_2_(SO_4_)_3_- (**C**) supplemented media. ^1^H,^1^H COSY (**D**) and ^1^H,^13^C HSQC (**E**) of the purified melanin by *S. nashvillensis* grown on CuSO_4_ plus Fe_2_(SO_4_)_3_ supplemented medium.

The melanin pigments produced in all the three best conditions (2.0 g∙L^−1^ of Fe_2_(SO_4_)_3_, CuSO_4_, or Fe_2_(SO_4_)_3_ plus CuSO_4_) were purified from the clarified broth supernatants through acidic precipitation at 4 °C, with recovery values ranging from 72.0 to 75.0%, and final pureness ranged from 79.7 to 82.3%. UV-visible spectra were acquired for each sample and compared to the spectrum of the melanin produced on the control medium (Figure 4). All the purified melanins showed similar absorption profiles, exhibiting the typical monotonic decay over the entire UV-visible range, with a maximum peak at 220 nm, similar to the control and to previously reported data [34] (Figure 4). FT-IR analyses of all the purified samples showed thirteen signals specific for the melanin pigment, most of them like the control (Figure 5A,B). First, a strong, broad peak centered between 3464 and 3383 cm^−1^ indicated the stretching of -OH and -NH groups of the indolic and pyrrolic rings (Peak 1), while the small, weak bands (Peak 2 and 3) between 2920 and 2969 cm^−1^ and between 2851 and 2853 cm^−1^ were attributed to the stretching vibration of aliphatic C-H groups. Two signals were noted for the control sample at around 2363 and 2338 cm^−1^ (Peaks 4 and 5), while only one was visible in Fe_2_(SO_4_)_3_- or in Fe_2_(SO_4_)_3_ plus CuSO_4_- supplemented samples (2346 and 2348 cm^−1^, respectively), as if the iron ions present in the medium partially bounded the melanin -O-H and -N-H groups, thus shielding some stretching vibrations of the amine, amide, or carboxylic acid groups of the indolic units (Figure 5A,B). These two signals were completely shielded in the case of CuSO_4_-supplemented samples. The signals between 1707 and 1745 cm^−1^ (Peaks 6) were attributed to the C=O stretching of quinone or carboxylic acid groups, while the peaks between 1647 and 1641 cm^−1^ (Peak 7) and 1537 and 1542 cm^−1^ (Peak 8) were usually due to the stretching of the aromatic C=C groups and to the bending of secondary NH groups, respectively (Figure 5A,B). This last peak was shown to be more intense in the case of CuSO_4_ and Fe_2_(SO_4_)_3_ plus CuSO_4_-supplemented samples than the control. The two signals between 1398 and 1476 cm^−1^ (Peak 9 and 10) were attributed to the -CH_2_-CH_3_ bending, which is usually considered to be characteristic of melanin pigments. The small peaks between 1231 and 1237 cm^−1^ and between 1153 and 1167 cm^−1^ (Peaks 11 and 12) were due to the stretching of phenolic groups, while peaks in the range between 1051 and 1077 cm^−1^ (Peak 13) were due to the bending of in-plane aliphatic and aromatic CH groups, respectively, and they are also considered characteristic of the melanin pigment (Figure 5A,B). All the purified melanins were also structurally characterized by mono- and bidimensional NMR analyses, confirming the eumelanic nature of the pigment (Figure 6). In detail, in the ^1^H NMR spectra, a similar pattern of signals was evident in the aromatic protons region of the three samples grown on the Fe_2_(SO_4_)_3_ (A)-, CuSO_4_ (B)- or CuSO_4_ plus Fe_2_(SO_4_)_3_ (C)-supplemented media, with signals at 6.62 and 7.2–7.4 p.p.m. ascribable to protons of the indolic ring along with a broad signal at 11.3 p.p.m., indicative of protons of indolic -NH groups. The assignment of the signals of the indolic protons was also supported by ^1^H,^1^H COSY (D) and ^1^H,^13^C HSQC (E) spectra. These signals were like the signals previously reported for the melanin pigment obtained when the bacteria was grown on the simple GEM III N medium [34].

### 2.3. Fermentation Experiments in Stirred Tank Reactor

**Figure 7:**
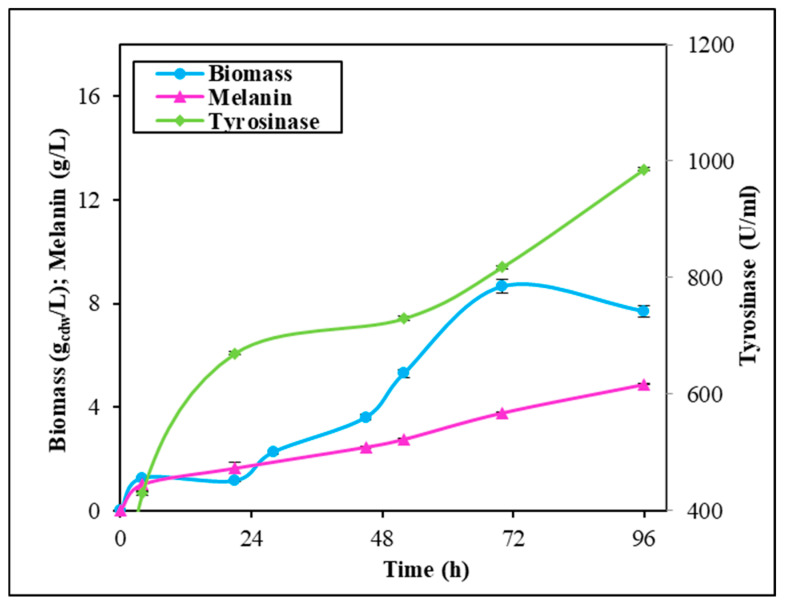
*S. nashvillensis* growth, melanin production, and tyrosinase activity in 96 h fermentations in STR on GEM III N medium supplemented with 2.0 g∙L^−1^ CuSO_4_ plus Fe_2_(SO_4_)_3_.

Given the observed synergistic effect of the 2.0 g∙L^−1^ Fe_2_(SO_4_)_3_ plus CuSO_4_ supplementation on melanin production, additional experiments were conducted under controlled conditions in STRs. These experiments aimed to potentially further enhance melanin production by implementing more rigorous control over the growth parameters (Figure 7). In these conditions, the microbial growth reached the maximum earlier than in shake flasks and 1.2 times higher, with a value of 8.7 ± 0.1 g_cdw_∙L^−1^ at 72 h. The addition of Fe_2_(SO_4_)_3_ plus CuSO_4_ also prompted melanin production that reached a maximum of 4.9 ± 0.1 g∙L^−1^ in 96 h, again 1.2 times higher than in shake flasks, with a yield on biomass of 0.56 ± 0.02 g∙g_cdw_^−1^ and a productivity of 0.051 ± 0.001 g∙L^−1^∙h^−1^ (Figure 7). Tyrosinase activity, instead, greatly increased, with a value of about 429.0 U∙mL^−1^ since the 4 h of growth to 986.0 U∙mL^−1^ at 96 h.

## 3. Discussion

*Streptomyces* strains might represent valid bacteria cell factories for the biotechnological production of microbial melanin, as they naturally synthesize this pigment as an extracellular product to protect themselves from stressful environmental conditions, such as persistent UV and visible light irradiation, extreme temperatures, drought, and metal exposure. As with other secondary metabolites, pigment synthesis might be driven by nutrient factors, such as the presence in the growth medium of metals or of diverse carbon and nitrogen sources or polyamines and oxygen availability [4,7,8,9,35]. To set up industrially reliable biotechnological procedures to manufacture melanin by using Streptomycetes, high production yields and innovative strategies of purification are needed [8]. A wise strategy for boosting pigment synthesis might be the supplementation of metal salts to the growth medium as the metal exposure triggers *Streptomyces* cells to produce more and more melanin. Incredibly, this approach has been poorly investigated so far. Only one paper added a single concentration of iron (II) or nickel (II) salts to the growth medium to specifically test their effects on *Streptomyces* sp. ZL-24 melanin production on agar plates [33]. The authors also set up the best pH and temperature conditions (the best values were pH 7.0 and 30 °C) for the strain, the optimal carbon and nitrogen sources, and the salt composition of the medium, thus reaching a maximum of 4.2 g∙L^−1^ soluble melanin after 120 h of growth on agar plates [33]. Different from what has been reported before, in this paper, for the first time, the effect of single supplementations or of coupled additions of different concentrations of Fe_2_(SO_4_)_3_ or CuSO_4_ was tested on the melanin production of *Streptomyces nashvillensis* DSM40314 in a liquid medium, first in shake flasks and then in STR fermentations, in 96 h runs. The addition of Fe_2_(SO_4_)_3_ did not affect the growth but greatly boosted melanin production, which reached a value of 1.71 ± 0.01 g∙L^−1^ when 2.0 g∙L^−1^ were added, which was 2.3-fold higher than the control. The addition of 2.0 g∙L^−1^ CuSO_4_, instead, boosted both the biomass and the melanin biosynthesis up to 2.53 ± 0.01 g∙L^−1^, which was 3.4-fold higher than the control, but it was the additive effects, due to the contemporary addition of 2.0 g∙L^−1^ Fe_2_(SO_4_)_3_ plus CuSO_4_, that greatly increased the production up to 4.04 ± 0.01 g∙L^−1^ in 96 h. This melanin concentration value was comparable to the one reported for *Streptomyces* sp. ZL-24, but it was obtained in less time, 96 h instead of 120 h. Moreover, in our case, the pigment was produced in a liquid medium, a growth strategy that might be easily scaled up and applied in real industrial processes [33]. In other literature studies, melanin concentrations in the range of the g∙L^−1^ scale were also reached in shake flask growths in the case of *Streptomyces djakartensis* NSS-3 (11.8 g∙L^−1^ of melanin) and in the case of *S.* 7VPTS-SR (5.5 g∙L^−1^) when grown on a medium containing peptone, proteose peptone, yeast extract, and mainly ferric ammonium citrate as salt [31,36] or, in the case of *S.* BJZ10 and of *S. kathirae* (3.0 and 13.7 g∙L^−1^, respectively), on media containing soluble starch, casein, and FeSO_4_·7 H_2_O between the salts, or amylodextrine, yeast extract, and CuSO_4_ and L-tyrosine, respectively [25,37]. The enhanced production obtained in this study perfectly correlated with the fact that tyrosinase was always higher than the control but with different trends depending on the type of supplemented salt. CuSO_4_ addition greatly boosted extracellular tyrosinase activity in the first 48 h compared with the control, while Fe_2_(SO_4_)_3_ presence in the medium had a prolonged, increased effect on tyrosinase activity during the whole run. So, when the two salts were contemporarily added, a constant, high tyrosinase action was observed during the whole growth. Given that tyrosinase activity represents a rate-limiting step in the melanin biosynthesis pathway [25,33], the re-gulation of this enzyme activity through the provision of suitable metal co-factors and the optimization of the aeration conditions during fermentations may further enhance its functioning and subsequently boost melanin production. This effect has been previously observed in the case of *S. roseochromogenes* [23], suggesting that well-regulated aeration in fermentation vessels could yield similar positive outcomes. In fact, in this study, the integration of dual-metal supplementation and continuous aeration resulted in a remarkable increase in tyrosinase activity, approximately 89 times greater than previously reported data in shake flask experiments. Although the melanin increase was not proportional, the final concentration of 4.9 g∙L^−1^ of the pigment significantly surpasses many melanin levels reported in the scientific literature. This value was 14 times greater than the melanin produced by *Streptomyces glaucenscens* NEAE-H (0.350 g∙L^−1^) over 168 h of growth [21] and 42 times higher than the pigment produced by *Streptomyces cavourensis* RD8 (0.116 g∙L^−1^), also determined after 168 h [20]. All the pigments produced in the different supplementation conditions were then also purified and characterized. The purification procedure took advantage of the extracellular nature of the melanin, resulting in high purification and recovery yields and in being very easy to apply, even if the pigment was produced in a medium containing metal ions. UV analyses revealed that all the melanin produced in the different conditions had the same maximum wavelength of absorbance (220 nm) as the control. This value of ma-ximum absorbance agrees with the wavelengths of other melanin pigments produced by other strains, like *Streptomyces lusitanus* DMZ-3 (that showed maximum between 200 e 300 nm) [22], *Streptomyces glaucenscens* NEAE-H (250 nm) [21], and *Streptomyces kathirae* SC-1 (220 nm) [25]. Instead, differences were noted in the profile of FT-IR analyses in the case of the melanin produced with Fe_2_(SO_4_)_3_- or in Fe_2_(SO_4_)_3_ plus CuSO_4_-supplemented samples. Only one of the two peaks generally attributed to the melanin O-H and N-H groups stretching vibrations were visible in these samples, as if the iron ions presence in the medium not only influenced tyrosinase activity and boosted melanin production but also partially interacted with the melanin structure throught weak bonds with these groups. Moreover, the NMR analyses confirmed the eumelanin-like structure of all the purified pigments.

## 4. Materials and Methods

### 4.1. Materials

Medium components like glucose, NaH_2_PO_4_·H_2_O, Na_2_HPO_4_, CuSO_4_, Fe_2_(SO_4_)_3_, and the polyethylene glycol (PEG) antifoam were purchased from Sigma-Aldrich (St. Louis, MO, USA), while yeast and malt extracts were purchased from Himedia Laboratories (Maharashtra, India). All the salts used for buffer preparation and then employed in biomass cell dry weight procedure for melanin purification or in the tyrosinase assay, as well as the synthetic melanin standard, were supplied by Sigma-Aldrich (St. Louis, MO, USA). L-DOPA for tyrosinase assay was purchased from TCI (Tokio, Japan). Chemicals for the melanin structural characterization by UV-visible absorbance, FT-IR, and NMR analyses were provided by Sigma-Aldrich (St. Louis, MO, USA) as well.

### 4.2. Microorganism and Media

*Streptomyces nashvillensis* DSM 40314 by DSMZ (Braunschweig, Germany) was grown and propagated on GYA medium [glucose (20.0 g∙L^−1^), yeast extract (20.0 g∙L^−1^), (NH_4_)_2_SO_4_ (2.0 g∙L^−1^), KH_2_PO_4_ (4.3 g∙L^−1^), and K_2_HPO_4_ (17.4 g∙L^−1^), at pH 7.0] and then stored at −80 °C in 20.0% (v/v) glycerol stock solutions, according to a previously reported procedure [34,37]. For the experiments, 100 µL of the bacteria glycerol stock solutions were used to inoculate each shake flask containing the GEM III N medium [glucose (12.0 g∙L^−1^), yeast extract (6.0 g∙L^−1^), malt extract (30.0 g∙L^−1^), NaH_2_PO_4_·H_2_O (5.8 g∙L^−1^), Na_2_HPO_4_ (8.2 g∙L^−1^), at pH 7.0] [33,34,37], and eventually different concentration of CuSO_4_ or/and of Fe_2_(SO_4_)_3_. All the media were first sterilized in an autoclave (ALFA-junior, VWR International PBI, Milan, Italy) at 120 °C for 20 min, without glucose, Na_2_HPO_4_, and CuSO_4_ or/and Fe_2_(SO_4_)_3_; they were then all singularly added later to the sterilized media as solutions of small volumes (2–4 mL) after being filtered with 0.22 µm membranes (Merck Millipore, Burlington, MA, USA).

### 4.3. Shake Flask Experiments

Experiments were always run in triplicate in 250 mL shake flasks containing 50 mL of the GEM III N medium at pH 7.0, 28 °C, and 250 rpm in a rotary air shaker (ISF-1-W, Kühner, Birsfelden (Basel), Switzerland) for 96 h [34]. The first runs were performed by supplementing different concentrations (6.25, 9.37, or 12.50 mM) of CuSO_4_ or different concentrations (6.58, 9.87, or 13.10 mM) of Fe_2_(SO_4_)_3_ in comparison to the control. The reported millimolar concentrations corresponded to 1.0, 1.5, and 2.0 g∙L^−1^ of each salt, respectively. Moreover, by supplementing NiCl_2_ to the medium, very small growth and no melanin production were noted, and no further experiments were performed with this metal. Further studies were carried out by adding to the medium 2.0 g∙L^−1^ of both CuSO_4_ and Fe_2_(SO_4_)_3_. In all the experiments, the microbial growth, melanin production, and tyrosinase activity were determined by withdrawing samples of broth (5 mL) at different time points. The biomass was determined as cell dry weight by filtering small volumes of the broth cultures (2 mL) on 0.22 μm polypropylene membranes (Merck Millipore, Burlington, MA, USA); these membranes were then washed with physiological saline solution and dried at room temperature up to the achievement of constant dry weights [19,20,21,22,23,24,25,26,27,28,29,30,31,32,33,34]. The remaining volumes of the samples were centrifuged at 4 °C and 5000 rpm for 20 min (Avanti J-20XPI, Beckman Coulter, Milan, Italy); then, the biomasses were discarded, and the supernatants were instead used to determine both melanin production and tyrosinase activity. To purify and characterize the melanin pigments obtained in the best supplementation conditions, 1 L shake flasks with 200 mL of the GEM III N medium (pH 7.0) supplemented with 2.0 g∙L^−1^ of CuSO_4_ and/or Fe_2_(SO_4_)_3_ were run in quadruplicate for 96 h, together with the control, in a rotary air shaker (ISF-1-W, Kühner, Birsfelden (Basel), Switzerland) at 28 °C and 250 rpm after being inoculated with 400 µL of bacterial glycerol stock solutions. Then, the broths were centrifuged for 20 min at 4 °C and 5000 rpm (Avanti J-20XPI, Beckman Coulter, Milan, Italy), and the clarified broth supernatants were used to purify the pigments.

### 4.4. Fermentation Experiments in Stirred Tank Reactor

Controlled fermentation experiments in a stirred tank reactor were performed in quadruplicate by using two MiniBio bioreactors (Applikon Biotechnology, Delft, The Netherlands) with 0.250 L vessels, with working volumes of 0.150 L (Applikon my-control, Gettingen, Germany). The fermentors were equipped with pH and temperature probes and four peristaltic pumps for the addition of alkali, acid, and, eventually, an antifoam solution. The pH controller unit delivered 30.0% NaOH and/or 30.0% H_2_SO_4_ solutions to keep the pH at the pre-set value of 7.0. The air stream was sterilized by filtration (cut-off 0.2 µm, Merck Millipore, Burlington, MA, USA) and continuously supplied to the reactor under controlled mass flow (1.1 vvm). A micro-sparger was used for optimal dispersion of the gas in the culture. The agitation speed was set to 400 rpm. The two vessels containing the GEM III N medium, without glucose, Na_2_HPO_4_, CuSO_4_, and Fe_2_(SO_4_)_3_, were sterilized in an autoclave. The other components were added to the medium before the inoculum as concentrated solutions after being filtered with 0.22 µm membranes (Merck Millipore, Burlington, MA, USA). Experiments were run for 96 h at 28 °C with 0.125 L of the GEM III N medium supplemented with 2.0 g∙L^−1^ of CuSO_4_ plus Fe_2_(SO_4_)_3_. During the experiments, the pH value was kept constant at 7.0 through the addition of 30.0% NaOH and/or 30.0% H_2_SO_4_ solutions; the stirring was kept constant at 400 rpm, while the airflow was at 1.1 vvm. The fermentation process parameters were remotely controlled and collected using a Digital Control Unit (DCU) (Applikon my-control, Gettingen, Germany). During the fermentation, broth samples (4 mL) were withdrawn to determine the cell dry weight, melanin production, and tyrosinase activity, as previously described.

### 4.5. Melanin Purification

The melanin pigments produced in 1 L shake flasks in the different supplementation conditions and the control were purified according to a two-step procedure [34]. First, the pigments were precipitated from the different supernatants (0.8 L each) by adding 5.0 M HCl, up to pH 1.5 [9,23]. The samples were kept overnight at 4 °C and then centrifuged at 4 °C and 5000 rpm for 20 min (Avanti J-20XPI, Beckman Coulter, Milan, Italy). All four collected, precipitated melanins were then washed three times with MilliQ water, centrifuged each time as previously described, and dried at room temperature. A second step of purification was then performed by washing all four samples again with the 5.0 M HCl solution under stirring conditions at room temperature for 24 h to eventually remove proteins and nucleic acids weakly bonded to the pigments, slightly modifying previously reported protocols [25,34,38]. The samples were then centrifuged at 4 °C and 4500 rpm for 20 min (Avanti J-20XPI, Beckman Coulter, Milan, Italy), and the melanins were then washed again three times with MilliQ water and dried as described above. Samples were withdrawn during the purification process to evaluate the final melanin pureness by performing UV-VIS analyses. In the end, all four final purified melanins were then characterized by UV-VIS, FT-IR, mono and bidimensional NMR analyses.

### 4.6. Melanin Determination by UV-Visible Analyses

The determination of the melanin concentrations in both shake flask and fermentation experiments, as well as of the final melanin pureness after the purification process, was performed using UV spectrophotometric analyses by measuring the absorbance at 220 nm (Spectrophotometer V-530, Jasco, Tokyo, Japan). After that, a calibration curve in the range from 0.0005 to 0.01 g∙L^−1^ was built by using the synthetic melanin standard [34]. To correctly monitor melanin production during the microbial growths, the absorbance measured for the supernatant samples at the initial time points (t 0 h, initial growth medium) was subtracted from the absorbance of samples at each different time point. Instead, the purity of the final purified melanins was determined after dissolving a defined amount of the dried samples in a defined volume of MilliQ water. Moreover, at the end of the downstream process, full UV-visible spectra, in the range from 190 to 400 nm, were acquired for all the purified melanin samples. All the standards and the samples were dissolved or diluted in 0.1 M NaOH solution, which was also always used as a blank.

### 4.7. Tyrosinase Activity Assay

The activity of extracellular tyrosinase, released during the bacterial growths, was assayed on aliquots of supernatant samples (1.0 mL) by using L-DOPA as substrate [39]. Small volumes (50 µL) of the samples were added to a freshly prepared solution (950 µL) of 2.0 mM L-DOPA in 13.0 mM KH_2_PO_4_ buffer at pH 6.5. The kinetic absorbance of the reaction mixture was acquired at 280 nm, at 25 °C, for 10 min (Spectrophotometer Jasco V-530, Jasco Europe, Cremella (LC), Italy). One unit of tyrosinase was defined as the amount of enzyme that gives an increase of 0.001 units of absorbance per min [39].

### 4.8. Melanin Structural Characterization

#### 4.8.1. Melanin Analysis by Fourier-Transform Infrared (FT-IR) Spectroscopy

The four purified melanin pigments (about 1.0–1.2 mg) were singularly mixed with KBr powder (198.0–199.0 mg) in an agate mortar, then ground and pressed to form translucent discs. The discs were scanned using Fourier-transform infrared spectroscopy (FT-IR) by using an FT-IR-4700 instrument (Jasco Europe, Cremella (LC), Italy), and spectra were recorded in duplicate in the 4000–700 cm^−1^ range by performing 600 scans, with a resolution of 2.0 cm^−1^.

#### 4.8.2. Melanin Analysis by Nuclear Magnetic Resonance (NMR) Spectroscopy

The purified melanin samples (35–40 mg) obtained from the supplemented media were dissolved in DMSO-*d*_6_ as a solvent and analyzed using NMR spectroscopy. ^1^H NMR spectra were recorded with a BrukerDRX-400 MHz instrument (Bruker Corporation, Billerica, MA, USA), whereas ^1^H,^1^H COSY and ^1^H,^13^C HSQC experiments were run at 400.1 MHz using standard pulse programs.

## 5. Data Analysis

All the data reported in this paper were the average values of at least three independent experiments, calculated with their standard deviations by using a Microsoft Office Excel 2007 program (Microsoft, Redmond, Washington, DC, USA).

## 6. Conclusions

In conclusion, in this paper, for the first time, it was demonstrated that melanin production by *Streptomyces nashvillensis* DSM40314 could be enhanced up to about 5.0 g·L^−1^ by wisely coupling a strategy of medium supplementation with iron plus copper salts, together with a STR fermentation approach that ensures strict, controlled aeration conditions. Furthermore, the pigments produced showed eumelanin-like structures, which are thus suitable for different industrial applications.

## Data Availability

The data presented in this study are available on request from the corresponding author.
